# Splenic tuberculosis presenting as fever of unknown origin with severe neutropenia

**DOI:** 10.1186/1476-0711-12-13

**Published:** 2013-06-18

**Authors:** Nahla A Azzam

**Affiliations:** 1 Division of Gastroenterology, Department of medicine, King Saud University, P.O. Box 2925(59), Riyadh, Saudi Arabia

**Keywords:** Splenic tuberculosis, Fever of unknown origin, Severe neutropenia

## Abstract

Fever of undetermined origin always poses a challenging problem to the physician. Tuberculosis is an important health problem in developing countries. It is mostly seen in immune-compromised patients. And it is one of the common causes of fever of unknown origin. I am reporting a case of a splenic tuberculosis in 48 years old male who is not known of any immune deficiency state, he was presented with 3 weeks history of fever and found to have severe neutropenia and with negative work up for all hematological, rheumatological and malignant causes. A computerized tomography scan of the abdomen confirmed splenic enlargement with multiples hypo dense lesions consist with either splenic infection or splenic lymphoma so exploratory splenectomy was performed. Histological examination revealed granulomatouse inflammation with numerous acid fast bacilli consist with tuberculosis. He was started on four anti-tuberculouse drugs. in less than one week his fever subside with normalization of his neutrophilic count

## Case presentation

The patient was a 48-year-old Syrian male with a 3 weeks history of remittent fever ranging between 37.0°C and 40.0°C. The fever was associated with chills, and was predominately at night with sweating. He also gave a history of 5 Kg weight loss along with loss of appetite. He had no other symptoms.

On admission, temperature was 39.4°C, pulse rate 140/min, respiratory rate 25/min and blood pressure was 95/50 mmHg. He looked pale. There was no lymphadenopathy. Abdominal Examination revealed palpable spleen 3 cm below costal margin. Chest and Heart were normal. Laboratory findings on admission showed: Hemoglobin level of 8.4 g/dL, Leucopenia with white blood cells (WBC) of 1.4/μL which further dropped to 0.5/μL (neutrophils 46%, lymphocytes 45%, monocytes 1%), an Erythrocyte Sedimentation Rate (ESR) of 90 mm/h. Renal and liver functions were normal. Chest roentgenograms consistently failed to demonstrate specific findings suggestive of tuberculosis. Bacteriologic cultures of blood, urine and cerebrospinal fluid were sterile. Further extensive studies for the fever origin turned out to be noncontributory for other infectious diseases, collagen diseases, malignancy or recognized immunodeficiency, including negative ANA, Anti-DNA, HIV, normal immunoglobulin level. Sputum cultures and stains for tubercle bacilli were negative. Bone marrow examination turned out normal with no evidence of hematological malignancy or any granuloma.

**Figure 1:**
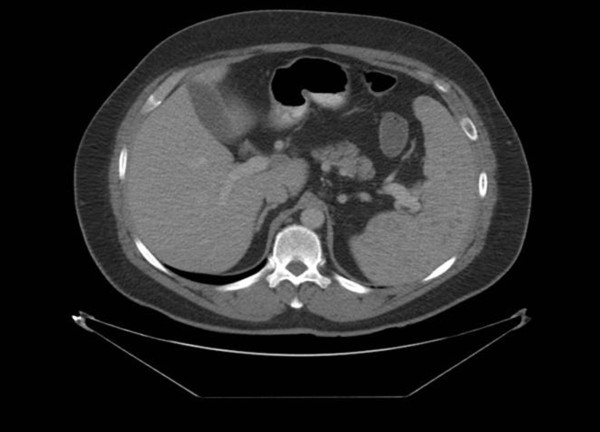
Multiple hypo dense lesion in the spleen.

**Figure 2:**
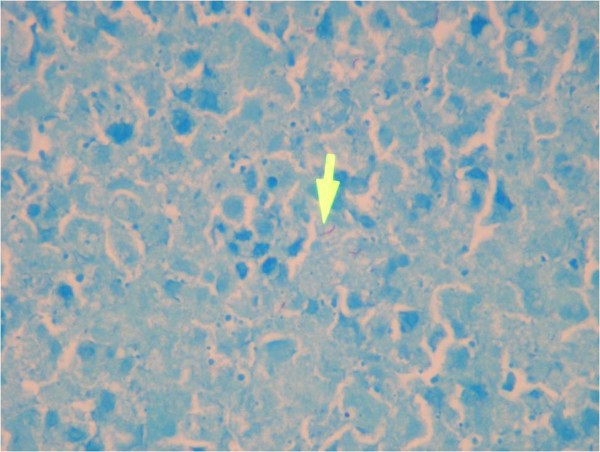
Zeil-Nelson stain showing acid fast bacilli (Arrow).

He was started on broad spectrum antibiotics without response to the fever, abdominal and chest Computerized Tomography (CT) was done which visualized splenomegaly with multiple hypo dense lesions consisting of either splenic lymphoma or tuberculosis. Rest of abdominal and chest organ were unremarkable (Figure 1). During this period, the purified protein derivative (PPD; 0.05 μg/0.1 mL) skin test yielded an erythematous and an induration of 4 mm. The patient remained sick with no improvement of his fever or leucocytes count which was below 0.5/μL. A thorough discussion was made with multidisciplinary team, and in view of highly suspected splenic lymphoma and poor diagnostic yield for fine needle aspiration from splenic nodules, it was decided that he undergoes laporatory splenectomy for diagnostic as well therapeutic purpose. Intra-operatively the spleen was enlarged 20 cm in length with multiple nodules found on the surface of the spleen. Ascites, however, was absent. Histological examination of the spleen showed that the basic lesion was caseating granuloma composed mainly of epithelioid cells and Langerhans giant cells. Ziehl–Neelsen staining was positive for tubercle bacilli (Figure 2).

The postoperative course was uneventful. The patient was placed on the four drug regimen (INH, RFP, PZA, ETM). From 3rd day post operative he was afebrile and well tolerating the Anti-TB medications. Eventually all of his laboratory results returned to normal, specifically WBC normalized to 5.2/μL. He received 12 months of Anti-TB medications.

The patient was then followed up for 6 months, and showed full recovery.

## Discussion

The patient reported in the present article presented with fever of unknown origin with severe neutropenia, and was diagnosed to have splenic tuberculosis based on echogenic findings of abdominal CT. The diagnosis was supported by histologic findings and he had eventual favorable clinical outcome following splenectomy along wth administration of antituberculous medication.

Splenic Tuberculosis is rare and develops as the result of either dissemination of pulmonary or biliary TB, following either ingestion of contaminated food or infected sputum. In developed countries it is seen in patients with HIV, but it is common health problem in developing countries with significant mortality and morbidity [1]. Our patient had isolated splenic tuberculosis, which is rare in immune-competent person [2].

The spleen is the most affected abdominal organ with tuberculosis [3]. In 1966, Lundstedt *et al.* reported 11 cases of splenic involvement in their series of 112 patients with abdominal tuberculosis [4]. Splenic enlargement was the most common manifestation in such cases [5]. Focal and hepatic involvement was also found to be frequently associated with this condition [5].

Splenic tuberculosis is generally a difficult diagnosis, as the clinical and laboratory findings are non specific. Although the ultrasound of abdomen is cheap and available but CT scan of abdomen is the investigation of choice which shows multiple rounded, hypo dense lesions in these cases, but such findings are also non specific since they may also be present with pyogenic splenic abscess or lymphomas [6].

Splenectomy has been advocated as the treatment of choice for splenic tuberculosis in the preantibiotic era. Splenectomy resulted in a recovery rate of approximately 60% [3]. Anti TB medication, however, should be considered as complementary therapy and should continue for 12 months.

Our patient case was peculiar since he had no immunosuppressive condition, and presented with fever for 3 weeks with sever neutropenia caused by splenic tuberculosis. To our knowledge such a case has not been published before, with the exception of one published case with neutropenia and splenic tuberculosis [7].

## Conclusion

Even if tuberculosis is a familiar condition, an unusual presentation may disguise its correct nature, expressing itself as cases of unexplained fever with severe neutropenia. Abdominal Tuberculosis should also be kept in mind in patients with fever, splenomegaly in TB endemic areas.

### Consent

Written informed consent was obtained from the patient for the publication of this report and any accompanying images.

## Competing interests

The author declare that I have no competing interests.
